# *PIK3CA*, *HRAS* and *PTEN* in human papillomavirus positive oropharyngeal squamous cell carcinoma

**DOI:** 10.1186/1471-2407-13-602

**Published:** 2013-12-17

**Authors:** Simion I Chiosea, Jennifer R Grandis, Vivian W Y Lui, Brenda Diergaarde, Jessica H Maxwell, Robert L Ferris, Seungwon W Kim, Alyssa Luvison, Megan Miller, Marina N Nikiforova

**Affiliations:** 1 Department of Pathology, University of Pittsburgh, 200 Lothrop St, Pittsburgh, PA 15213, USA; 2 Department of Otolaryngology, University of Pittsburgh, 200 Lothrop Street, Pittsburgh, PA 15213, USA

**Keywords:** Oropharyngeal squamous carcinoma, *PIK3CA*, *HRAS*, *PTEN*, HPV

## Abstract

**Background:**

Recent genomic evidence suggests frequent phosphatidylinositide 3-kinase (PI3K) pathway activation in human papillomavirus (HPV) positive oropharyngeal squamous cell carcinoma. Mutations/amplification of the gene encoding p110α catalytic subunit of phosphoinositide 3-kinase (*PIK3CA*)*,* loss of phosphatase and tensin homolog (*PTEN*) and *HRAS* mutations are known to activate PI3K pathway.

**Methods and results:**

*PIK3CA* mutations were identified by Sanger sequencing in 23 of 75 (31%) HPV-positive oropharyngeal carcinomas, including exon 9 (p.E545K [n = 10] and p.E542K [n = 5]) or exon 20 (p.H1047Y, n = 2) mutations. Five rare and one novel (p.R537Q) *PIK3CA* mutations were identified. *HRAS* mutation (p.Q61L) was detected in 1 of 62 tested cases. *PIK3CA* amplification by fluorescence *in situ* hybridization (FISH) was identified in 4 cases (4/21, 20%), while *PTEN* loss was seen in 7 (7/21, 33%) cases (chromosome 10 monosomy [n = 4], homozygous deletion [n = 3]).

**Conclusions:**

Overall, genetic alterations that likely lead to PI3K pathway activation were identified in 34 of 75 cases (45%) and did not correlate with disease specific survival. These findings offer a molecular rationale for therapeutic targeting of PI3K pathway in patients with HPV-positive oropharyngeal carcinoma.

## Background

The phosphatidylinositide 3-kinase (PI3K) pathway is activated in about half of head and neck squamous cell carcinomas (SCC) by a number of mechanisms, including mutation or amplification of the gene encoding p110α catalytic subunit of phosphoinositide 3-kinase (*PIK3CA)*[1-4]. The higher incidence of PI3K pathway activation in oropharyngeal SCC was previously reported [5]. Oropharyngeal SCC are increasingly associated with human papillomavirus (HPV) infection [6,7] and the higher prevalence of PI3K pathway abnormalities in these tumors was eventually linked to HPV [8,9].

Most recent characterization of the mutational landscape of head and neck SCC showed that the genetic profile of HPV-positive SCC is distinct from that of HPV-negative SCC. For instance, HPV-positive oropharyngeal SCC harbor fewer mutations overall (e.g., no TP53 mutations) and more *PIK3CA* mutations. Specifically, of the 15 HPV-positive SCC with known *PIK3CA* status reported in the literature, 4 tumors harbored *PIK3CA* mutation (4/15, 27%) [10,11]. In contrast, *PIK3CA* mutations are present in about 5% (5/91) of HPV-negative head and neck SCC. The higher incidence of *PIK3CA* mutations in HPV-positive SCC suggests a new therapeutic option, as PI3K pathway is targeted by multiple drugs in development: PX-866 [12], and MK-2066 [13], and RAD001 [14]. Indeed, our most recent findings demonstrated that HPV-positive SCC tumorgrafts with activating *PIK3CA* mutation were highly responsive to PI3K-targeted therapy [15].

Increased PI3K signaling can also result from mutations in other genes in the PI3K pathway such as *HRAS*[16,17]*.* In addition to *PIK3CA* mutations and/or amplification, PI3K pathway may also be activated due to phosphatase and tensin homolog (*PTEN*) deletion, a known negative regulator of the PI3K signaling pathway [18].

The aim of the present study was to elucidate the molecular basis for therapeutic targeting of PI3K pathway in HPV-positive oropharyngeal SCC by characterizing the prevalence and prognostic significance of *PIK3CA* and *HRAS* mutations, *PIK3CA* amplification, and *PTEN* loss in 75 patients with HPV-positive oropharyngeal SCC.

## Methods

### Patients

This study was approved by the Institutional Review Board of the University of Pittsburgh Medical Center (IRB# PRO11010195). Seventy five cases of HPV-positive oropharyngeal SCC were identified from 1983 to 2007 and satisfied the following inclusion criteria: availability of formalin fixed paraffin embedded tissue, p16 immunohistochemistry and HPV in situ hybridization positivity, presence of tumor areas with >50% represented by cancer cells, and extraction of adequate DNA.

### HPV in situ hybridization and p16 immunohistochemistry

HPV detection by in-situ hybridization was performed using probes targeting 37 distinct HPV subtypes, including 6, 11, 16, 18, 31, 33, 35, 39, 45, 51, and 52 (Y1404; Dako, Carpinteria, CA). Five-micrometer tissue sections were deparaffinized and digested with proteinase K (Roche Diagnostics, Indianapolis, IN). Cases with punctate nuclear signal were considered positive [19].

For p16 analysis, five-micrometer sections were deparaffinized. Heat-induced epitope retrieval was then performed in a citrate buffer. Immunohistochemistry for p16 (G175-405; BD Pharmingen, San Diego, CA) was performed as per the manufacturer’s protocol. Cases were considered positive if >70% of tumor cells showed diffuse and strong cytoplasmic and nuclear staining [19].

### PIK3CA and HRAS mutation analysis

Tissue cores from tumor targets were obtained as previously described [20]. DNA was isolated from tissue cores using the DNeasy tissue kit (Qiagen, Valencia, CA) according to the manufacturer’s instructions. For the detection of mutations, DNA was amplified with primers flanking exon 3 of the *HRAS* gene (forward primer 5′- GTC CTC CTG CAG GAT TCC TA -3′ and reverse primer 5′- CGG GGT TCA CCT GTA CT -3′), exon 9 of the *PIK3CA* gene (forward primer 5′- TGA AAA TGT ATT TGC TTT TTC TGT -3′ and reverse primer 5′- TGT AAA TTC TGC TTT ATT TAT TCC -3′) and exon 20 of the *PIK3CA* gene (forward primer 5′- TTT GCT CCA AAC TGA CCA A -3′ and reverse primer 5′- GCA TGC TGT TTA ATT GTG TGG -3′). PCR products were sequenced in both sense and antisense directions using the BigDye Terminator v3.1 cycle sequencing kit on ABI 3730 (Applied Biosystems, Inc., Foster City, CA) according to the manufacturer’s instructions (Additional file 1: Figure S1 and Additional file 2: Figure S2). The sequences were analyzed using Mutation Surveyor software (SoftGenetics, LLC., State College, PA).

The presence of most common *PIK3CA* mutations (p.E545K and p.E542K) was confirmed by SNaPshot PCR as per the manufacturer’s manual and as previously described [21] (Additional file 3: Figure S3). Briefly, primers for exon 9 (forward 5′-AGTAACAGACTAGCTAGAGA-3′ and reverse 5′-ATTTTAGCACTTACCTGTGAC-3′) and exon 20 (forward 5′-GACCCTAGCCTTAGATAAAAC-3′ and reverse 5′-GTGGAAGATCCAATCCATTT-3′) were used for amplification. Denatured products were analyzed on an ABI 3730 DNA analyzer (Applied Biosystems, Foster City, CA, USA).

### PTEN and PIK3CA fluorescence in situ hybridization (FISH)

Cases with known wild type *PIK3CA* and *HRAS* and available tissue were tested for *PIK3CA* and *PTEN* copy number changes by FISH (n = 22) (Additional file 4: Figure S4). Sixty to 130 cells were analyzed. *PTEN* (SpectrumOrange) and chromosomal enumeration probe 10 (CEP10, Spectrum Green) FISH was performed as per manufacturer’s recommendations (Abbot Molecular, Des Plaines, IL, USA) and as previously described [22]. Results were interpreted using previously established thresholds [23,24]: *PTEN* homozygous deletion was defined as >20% of cells without *PTEN* locus signal and the presence of ≥2 CEP10 signals. Hemizygous *PTEN* deletion was defined as >30% of cells with only one *PTEN* signal and the presence of ≥2 CEP10 signals. As previously suggested, cases with >50% of cells with a single CEP10 signal were categorized as “Chromosome 10 monosomy”, an additional mechanism of *PTEN* loss [22]. Despite repeated hybridization attempts, no data were obtained in one case.

*PIK3CA* (Spectrum Green) and CEP3 (Spectrum Orange) (Abbot Molecular, Des Plaines, IL, USA) FISH was performed as per manufacture’s recommendation. *PIK3CA* amplification was defined as *PIK3CA*/CEP3 >2. Despite repeated hybridization attempts, no data were obtained in one case.

### Statistical analysis

The primary endpoint was disease-specific survival (DSS) defined as time elapsed from the date of diagnosis until death from cancer. DSS was chosen as a primary endpoint over the overall survival due to the predominance of patients deceased from causes unrelated to the oropharyngeal SCC (n = 21). DSS was assessed only for patients with > 2 months of follow-up (n = 72). Patients who were alive at last follow-up or had died from other causes were censored. Survival data were presented as Kaplan-Meier plots. The log rank test was used to test survival equality. Covariates examined for association with survival included age, gender, smoking (ever versus never), site (tonsil and base of tongue), T and N classification, AJCC clinical stage, adjuvant chemotherapy or radiotherapy. Cross-tabulated categorical data were tested for independence with Fisher’s exact test.

## Results

**Figure 1:**
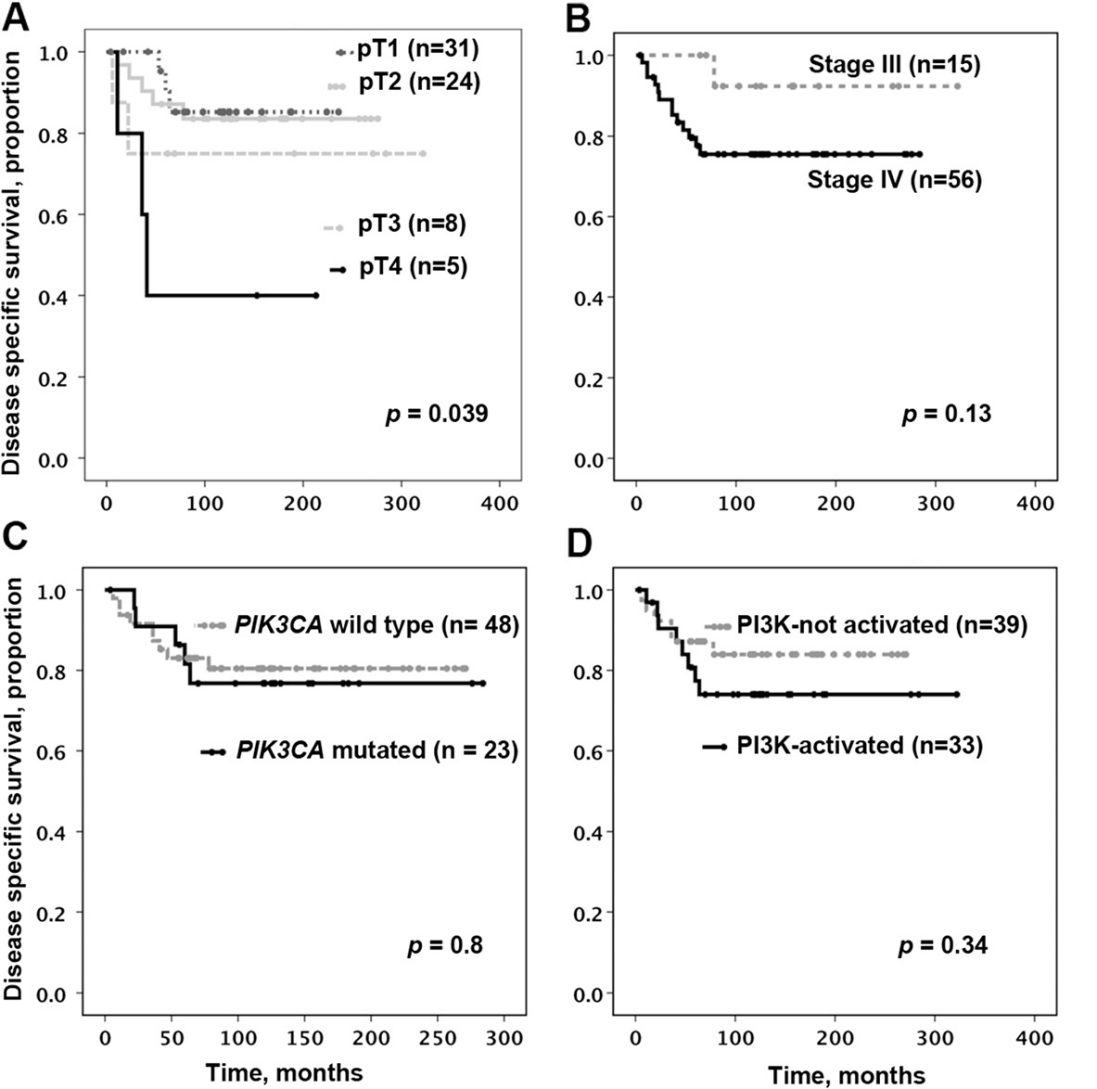
**Disease specific survival (DSS) of patients with p16 and human papillomavirus positive oropharyngeal squamous cell carcinoma.** DSS was assessed only for patients with > 2 months of follow-up (n = 72). **A**. pT stage and disease specific survival. **B**. Clinical stage and disease specific survival. **C**. *PIK3CA* mutational status and disease specific survival. **D**. Presumed activation of PI3K pathway and disease specific survival. Cases with PI3K pathway activating alteration with < 2 months of follow-up were excluded from the DSS analysis.

The clinicopathologic characteristics of 75 HPV-positive oropharyngeal SCC patients are summarized in Table 1. The mean follow-up was 122 months (minimum – maximum, 4–322). While 21 patients deceased of causes unrelated to oropharyngeal SCC, 14 patients died of disease. The 3-year disease specific survival (DSS) was 85%. In this clinicopathologically uniform group of patients only pathologic tumor stage (pT) correlated with DSS (Figure 1A). For instance, the DSS at 5 years was 87% for pT1 and 40% for pT4 (p = 0.039). There was a trend toward a worse DSS for patients with clinical stage IV disease; however, it did not reach statistical significance, most likely due to the low number of events in stage III patients (Figure 1B). It is noteworthy that patients’ gender, age, smoking history, oropharyngeal sub-site, pN, and treatment modality was not associated with survival in these individuals.

**Table 1:** **Clinicopathologic features of patients with human papillomavirus positive oropharyngeal SCC, overall and by *PIK3CA *** **mutational status**

**Feature**		**Total**^**1**^**, n = 75**	***PIK3CA *** **wild type**	***PIK3CA *** **mutant**	**Notes**
			**n = 51**	**n = 23**	
Men/women		64/11	43/8	20/3	n.s.
Average age, years (range)		56 (34–78)	56	55.3	n.s.
Smoking^2^	Never	22	15	7	n.s.
	Ever	40	26	14	
Site^3^	BOT	31	24	7	n.s.
	Tonsil	43	27	15	
pT^4^	1	34	23	11	n.s.
	2	25	17	8	
	3	7	4	3	
	4	5	4	1	
pN^6^	0	2	2	0	n.s.
	1	16	11	5	
	2	51	35	15	
	3	5	3	2	
Resection Margins^5^	Positive	10	7	3	n.s.
	Negative	54	36	18	
Clinical Stage^6^	I	1	1	0	n.s.
	III	15	10	4	
	IV	58	40	18	
Therapy^7^	RT	43	32	11	n.s.
	CRT	21	14	7	
Non-keratinizing		52	35	17	n.s.
Mixed		23	17	5	
Deceased of disease		14	9	5	

^1^For one patient with *HRAS* mutation *PIK3CA* status was indeterminate. The clinicopathologic features of the case with indeterminate *PIK3CA* status are not presented in the columns 3 and 4 of the Table.

^2^Smoking history was not available for 13 patients.

^3^The oropharyngeal sub-site for one case was unknown.

^4^pT was unknown for 4 cases with biopsies only.

^5^Margin status was not assessed in 11 cases (biopsies, fragmented specimens).

^6^pN and clinical stage were unknown for 1 patient.

^7^Seven patients received no postoperative therapy; for 4 patients these data were not available.

*BOT* base of tongue, *RT* radiotherapy, *CT* chemotherapy, *CRT* chemoradiotherapy, *mixed* keratinizing and non-keratinizing, *n.s.* not statistically significant.

### *PIK3CA, HRAS,* and *PTEN* alterations

*PIK3CA* mutations were identified in 23 of 75 patients with oropharyngeal SCC (31%), including exon 9 (p.E545K [n = 10] and p.E542K [n = 5]) or exon 20 (p.H1047Y, n = 2) mutations. Five cases with rare mutations and one case with novel mutation are presented in Table 2. Patients’ gender, age, smoking history, oropharyngeal sub-site, pT, pN, clinical stage, and treatment modality were similar between cases with wild type and mutated *PIK3CA*. Disease specific survival (DSS) of the patients in these two groups was not significantly different (Figure 1C).

**Table 2:** **Rare and novel *PIK3CA *** **mutations in HPV positive oropharyngeal SCC**

**Mutation**	**Clinicopathologic features and number of cases in this study, n**	**Prior reports and References**
Exon 9, c.1571G > A, p.R524K and c.1573G > A, p.E525K	53 year old man, NK, pT1 N2, no postoperative therapy, NED at 132 mo, n = 1	None. While individually both mutations were previously reported, the combination of the two was not.
Exon 9, c.1610G > A, p.R537Q	60 year old man, NK, pT1 N2, CRT, DOD in 22 months, n = 1	None
Exon 20, c.2975G > A, p.R992Q	68 year old man, NK, pT2 N1, radiotherapy, DOC at 70 months, n = 1	Glioblastoma multiforme, n = 1 [25]
Exon 20, c.3103G > A, p.A1035T	54 year old man, mixed K& NK pT2 N2, radiotherapy, DOD at 53 months, n = 1	Endometrial adenocarcinoma, n = 1 [26]
		Urothelial carcinoma of the bladder, n =1 [27]
Exon 20, c.3129G > C,	48 year old man, mixed K & NK, pT3 N1, radiotherapy, NED at 284 months, n = 1	Carcinoma of the breast, n = 2 [28]
p.M1043I		
Exon 20, c.3153G > A, p.W1051*	71 year old man, NK, pT1 N1, treated with radiotherapy, DOC at 182 months, n = 1	Ovarian clear cell carcinoma, n = 1; conventional colorectal carcinoma, n = 1; [29,30]

*NK* non-keratinizing, *K* keratinizing, *DOC* deceased of other causes, *DOD* deceased of disease, *CRT* chemoradiotherapy, *NED* no evidence of disease.

*HRAS* mutation (c.182A > T, p.Q61L) was identified in 1 of 62 tested cases (or in 1 of 58 successfully tested cases; in 4 cases the status of *HRAS* was indeterminate). In the only case with *HRAS* mutation, the mutational status of *PIK3CA* was indeterminate.

*PIK3CA* amplification was identified in 4 of 21 cases (20%). *PTEN* loss was identified in 7 of 21 cases (33%) (chromosome 10 monosomy [n = 4), homozygous deletion [n = 3]; note, for one of the cases with homozygous deletion clinical follow-up was not available).

Assuming that *PIK3CA* mutation or amplification, *HRAS* mutation, or loss of *PTEN* lead to PI3K pathway activation, patients with tumors harboring one of these events were combined into a “PI3K-activated” group and compared to patients whose tumors did not harbor any of the above genetic alterations. PI3K pathway activation did not correlate with DSS (Figure 1D).

## Discussion

The clinical and pathologic characteristics of our HPV-positive oropharyngeal SCC population and the performance of conventional pathologic prognosticators (e.g., pT, pN) are consistent with prior reports [31].

To our knowledge, this is the largest HPV-positive oropharyngeal SCC cohort to undergo evaluation for *PIK3CA* and *HRAS* mutation and *PIK3CA* and *PTEN* amplification/loss. Our findings suggest that mutation or amplification of *PIK3CA* may represent the most common alteration in HPV-positive oropharyngeal SCC. It is noteworthy that recent mutational analyses of head and neck SCC also found *PIK3CA* alterations, albeit at lower rates [10,11,15]. The variation in *PIK3CA* mutation incidence is most likely due to the relative underrepresentation of HPV-positive oropharyngeal SCC in other cohorts, use of oropharyngeal site as a surrogate marker for HPV status, and the use of different techniques to assess for *PIK3CA* mutations. The recently published data [11,15] highlighted an interesting phenomenon that even though HPV-positive SCC harbored fewer mutations on average, as high as 20% of HPV-positive SCC (3/15 cases [15]) harbored *PIK3CA* mutation as the only cancer gene mutation, indicating that PI3K pathway mutations are enriched in HPV-positive tumors despite the lower rate of gene mutations in general. The higher prevalence of PI3K pathway abnormalities in oropharyngeal SCC was previously linked to HPV [8,9].

All mutations found in the samples of HPV-positive oropharyngeal SCC were heterozygous with mutant allelic frequency that appeared to range from 20% to 50% of alleles (corresponding to 40%– 100% of cancer cells with a heterozygous mutation). None of the cases showed mutant allelic frequency of more than 50% suggesting that loss of the wild type *PIK3CA* allele or amplification of the mutant *PIK3CA* allele in cancer cells is exceedingly rare.

Although *HRAS* mutations have been reported to modulate signaling through the PI3K pathway [32,33], the role of the mutation found in a single HPV-positive oropharyngeal SCC in this study remains unclear.

*PTEN* is generally understood to function as a tumor suppressor gene and to negatively regulate *PI3K* pathway. Therefore, loss of *PTEN* should lead to PI3K pathway activation. The incidence of *PTEN* alterations in head and neck SCC varies in the literature and there is little indication that *PTEN* loss has an independent prognostic value [34,35]. We found that *PTEN* loss (as assessed by FISH) was relatively common in HPV-positive oropharyngeal SCC.

Activation of the PI3K pathway, generally by virtue of *PIK3CA* gene amplification, has been previously reported to represent a poor prognostic biomarker in head and neck SCC [36]. Others have reported that phosphorylation of AKT, a downstream target of PIK3CA, is associated with poor clinical outcome in oropharyngeal SCC, specifically [37]. Although HPV status was not specifically assessed in this cohort of oropharyngeal SCC, it is reasonable to presume that it was enriched for HPV-positive SCC. Our analysis showed no association between the genetic alterations we assessed for (combined into a “PI3K activated” group) and clinical outcome. Prior reports have generally focused on a single alteration or biomarker assessment. It is possible that some of the alterations we detected in HPV-positive oropharyngeal SCC do not activate the pathway as predicted. Or, more likely, each alteration modulates PI3K oncogenic signaling. Further functional studies in relevant preclinical models are needed to decipher the precise contribution of each mutation, amplification and/or loss to PI3K pathway status in HPV-positive oropharyngeal SCC.

One of the technical limitations of this study is that we restricted our assessment to exons 9 and 20 of *PIK3CA* gene and we have likely underestimated the frequency of *PIK3CA* mutation in this cohort. Similarly, we only assessed codon 61 of *HRAS* and did not perform codon 12/13 testing. Therefore, the actual mutation frequency of both *PIK3CA* and *HRAS* could be higher than reported here.

The variety of potential mechanisms leading to PI3K pathway activation underscores the complexity of the potential implications of our findings. It is possible, as reported by others and us, that head and neck SCC harboring “driver” *PIK3CA* mutations demonstrate enhanced response to PI3K pathway inhibitors [15,38,39]. Similar findings have been reported in clinical trials of patients with breast or gynecologic malignancies [40]. PI3K pathway inhibitors are under early investigation in head and neck SCC and clinical results are not yet available.

The EGFR monoclonal antibody cetuximab is FDA-approved in both newly diagnosed head and neck SCC as well as in the recurrent or metastatic setting [41]. We previously reported that PI3K pathway activation correlates with clinical resistance to cetuximab in head and neck SCC patients and targeting the PI3K pathway enhanced the antitumor effects of EGFR inhibitors in head and neck SCC preclinical models [42-44]. Therefore, molecular determinants of PI3K activation may identify individuals who may benefit from co-targeting of EGFR in conjunction with PI3K pathway inhibition.

## Conclusion

In conclusion, we report an analysis of a large HPV-positive oropharyngeal SCC cohort and demonstrate distinct, but perhaps functionally homologous, mechanisms of PI3K pathway activation: *PIK3CA* mutations/amplification, *HRAS* mutation, or *PTEN* loss. We provide evidence, for the first time, of potentially activating genetic alterations of the PI3K signaling pathway in about 45% (34/75) of HPV-positive oropharyngeal SCC. The significance of the affected *PIK3CA* exon or specific *PIK3CA* mutation types, mechanism of *PTEN* loss, and the association with alternative mechanisms of PI3K signaling remain incompletely understood. Our findings offer a molecular basis for future studies of therapeutic targeting of PI3K pathway in HPV-positive oropharyngeal SCC.

## Competing interests

P50CA97190 and the American Cancer Society (to JRG) and the Patricia L. Knebel Fund of the Pittsburgh Foundation (to VWYL).

## Authors’ contributions

MM and AL carried out molecular genetic studies. SC, JRG, VWYL drafted the manuscript. SC, MNN conceived, designed the study, and acquired the data. SC performed the statistical analysis. All authors were involved in analysis and interpretation of data and critical revision of the manuscript. All authors read and approved the final manuscript.

## Pre-publication history

The pre-publication history for this paper can be accessed here:

http://www.biomedcentral.com/1471-2407/13/602/prepub

## Supplementary Material

Additional file 1: Figure S1 Representative cases of human papillomavirus-positive oropharyngeal squamous cell carcinoma, with *HRAS* (A) or *PIK3CA* mutations (B through I), sequencing electropherograms (SE). Portions of the SE surrounding the point mutation were scanned. A. *HRAS* c.182A>T, p.Q61L. B. *PIK3CA*, exon 9, c.1571G>A, p.R524K and c.1573G>A, p.E525K. C. *PIK3CA*, exon 20, c.3103G>A, p.A1035T. D. *PIK3CA*, exon 20, c.3129G>C, p.M1043I. E. *PIK3CA*, exon 20, c.3139C>T, p.H1047Y. F. *PIK3CA*, exon 20, c.3153G>A, p.W1051*.G. *PIK3CA*, exon 9, c.1633G>A, p.E545K. H. *PIK3CA*, exon 9, c.1610G>A, p.R537Q. I. *PIK3CA*, exon 20, c.2975G>A, p.R992Q. Click here for file

Additional file 2: Figure S2 Representative sequencing electropherograms (SE) illustrating *PIK3CA* mutations. The entirely scanned SE are presented to illustrate low background. Click here for file

Additional file 3: Figure S3 SNaPshot detection of common hotspot mutations in the exon 9 of the *PIK3CA* gene. SNaPshot result illustrating c. 1633G>A, p.E545K electropherogram pattern (mutant peak is indicated by a grey arrow). The bases are color-coded: “A” – green, while “G” – blue. The red trigon-shaped peaks represent internal size standards. Click here for file

Additional file 4: Figure S4 Representative images of *PTEN* and *PIK3CA* fluorescence in situ hybridization, FISH, original magnification 1000x. All cases included in this figure are oropharyngeal squamous cell carcinoma that are human papillomavirus-positive, *HRAS* wild-type, *PIK3CA* wild-type, without *PIK3CA* amplification. The nuclei were counterstained with DAPI/Antifade 1 (blue) (Vysis, Inc., Downers Grove, IL). A. *PTEN* FISH, representative field: most nuclei are characterized by two *PTEN* signals (orange) and two chromosomal enumeration probe 10 (CEP10) signals (green), consistent with normal *PTEN* copy number. B. *PTEN* FISH, representative field: 57.1% of cells (36/63) showed only one pair of *PTEN* and CEP10 signals, consistent with chromosome 10 monosomy. C. *PTEN* FISH, representative field: 90.2% (55/61) of analyzed cells showed no *PTEN* signal, consistent with homozygous *PTEN* loss. Also, 55.7% (34/61) of analyzed cells showed only one CEP 10 signal consistent with chromosome 10 monosomy. The *PTEN*/CEP10 ratio was 0.11. D. *PIK3CA* (green) and CEP3 (orange) FISH, representative field. The *PIK3CA/*CEP3 ratio is 2.5. Click here for file
