# Supplementary figures and images for: PIK3CA, HRAS and PTEN in human papillomavirus positive oropharyngeal squamous cell carcinoma
Source: BMC Cancer. 2013 Dec 17;13:602. doi: 10.1186/1471-2407-13-602 (PMC3878565; doi:10.1186/1471-2407-13-602)

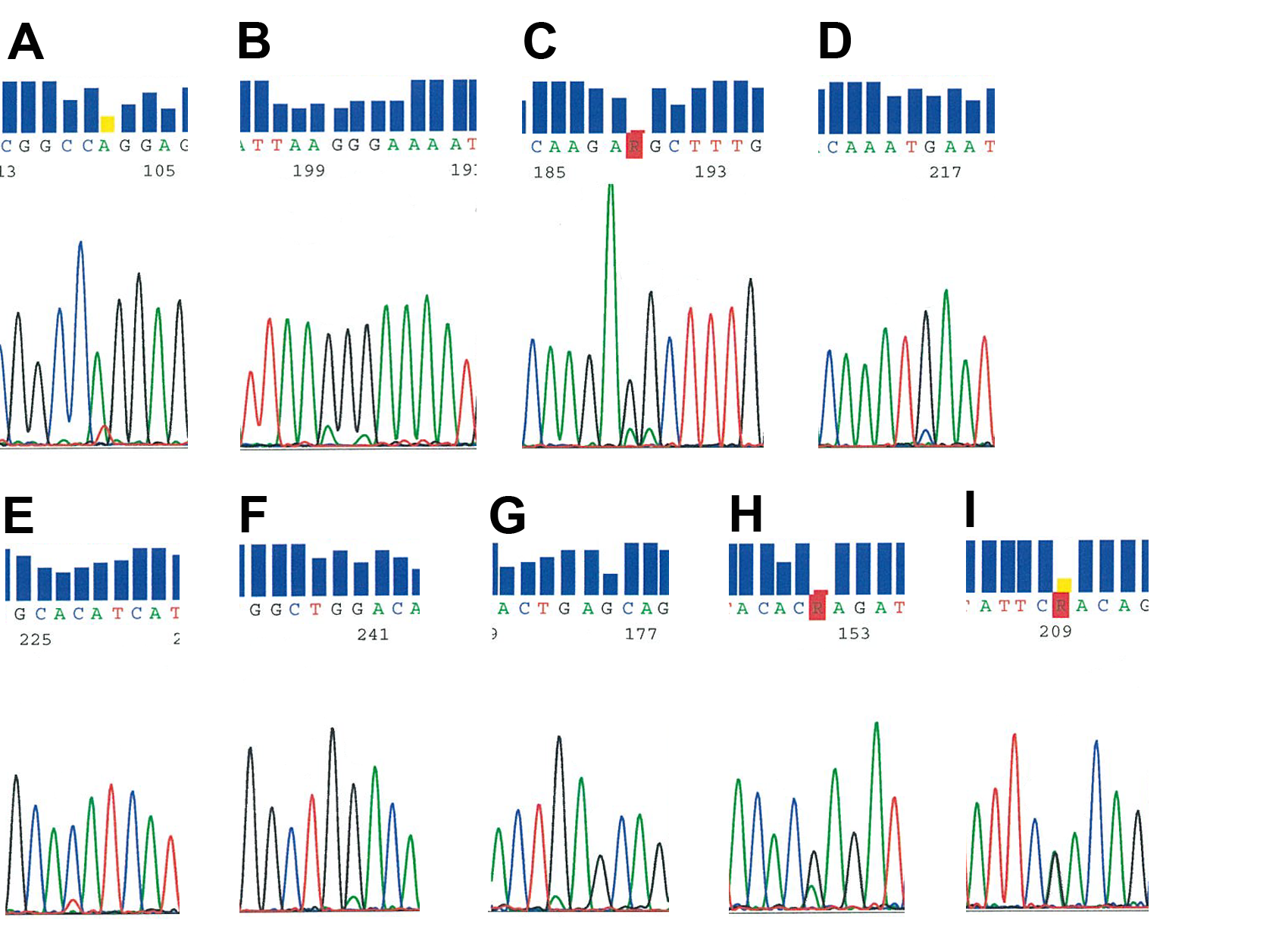

Supplement: Additional file 1: Figure S1 — Representative cases of human papillomavirus-positive oropharyngeal squamous cell carcinoma, with HRAS (A) or PIK3CA mutations (B through I), sequencing electropherograms (SE). Portions of the SE surrounding the point mutation were scanned. A. HRAS c.182A>T, p.Q61L. B. PIK3CA, exon 9, c.1571G>A, p.R524K and c.1573G>A, p.E525K. C. PIK3CA, exon 20, c.3103G>A, p.A1035T. D. PIK3CA, exon 20, c.3129G>C, p.M1043I. E. PIK3CA, exon 20, c.3139C>T, p.H1047Y. F. PIK3CA, exon 20, c.3153G>A, p.W1051*.G. PIK3CA, exon 9, c.1633G>A, p.E545K. H. PIK3CA, exon 9, c.1610G>A, p.R537Q. I. PIK3CA, exon 20, c.2975G>A, p.R992Q. [file 1471-2407-13-602-S1.tiff]

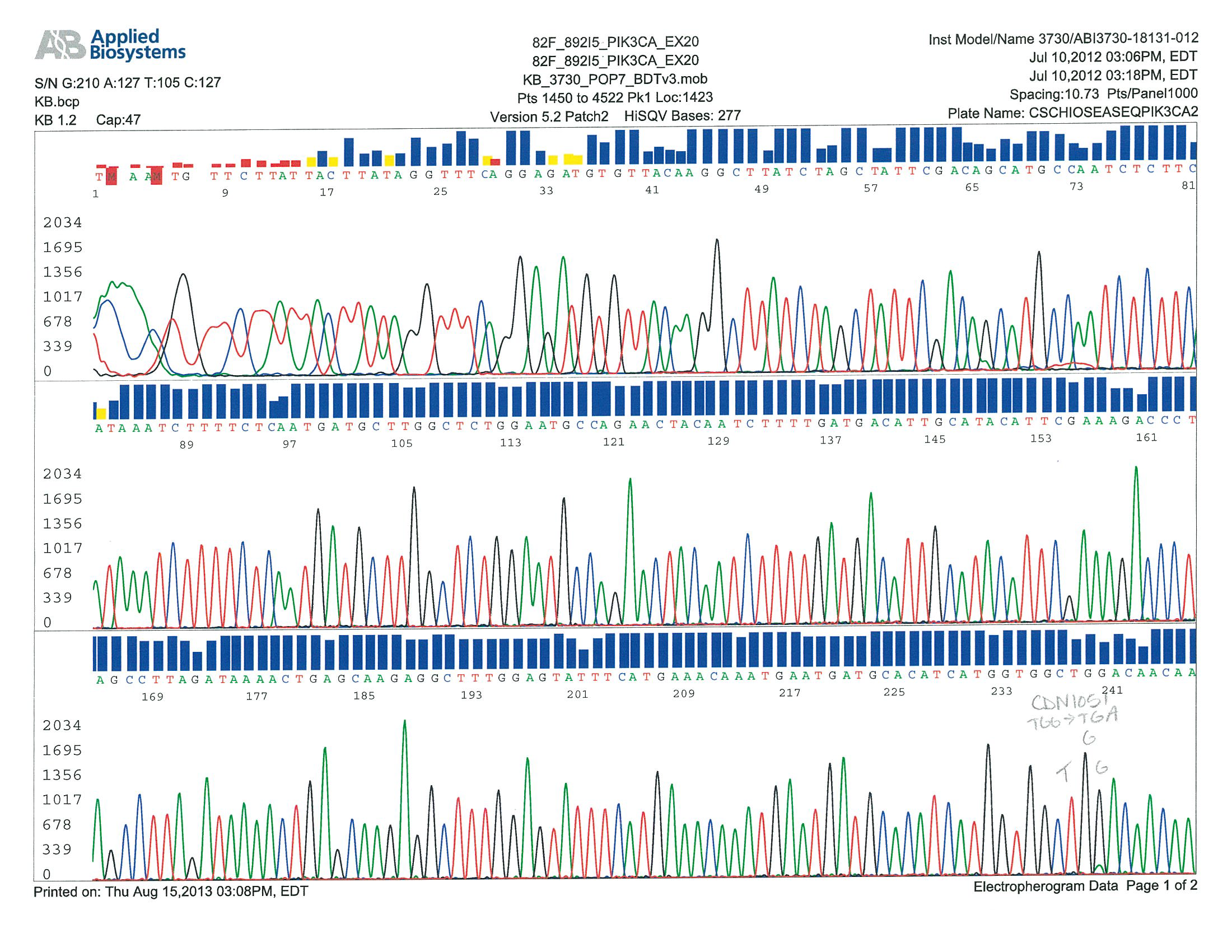

Supplement: Additional file 2: Figure S2 — Representative sequencing electropherograms (SE) illustrating PIK3CA mutations. The entirely scanned SE are presented to illustrate low background. [file 1471-2407-13-602-S2.tiff]

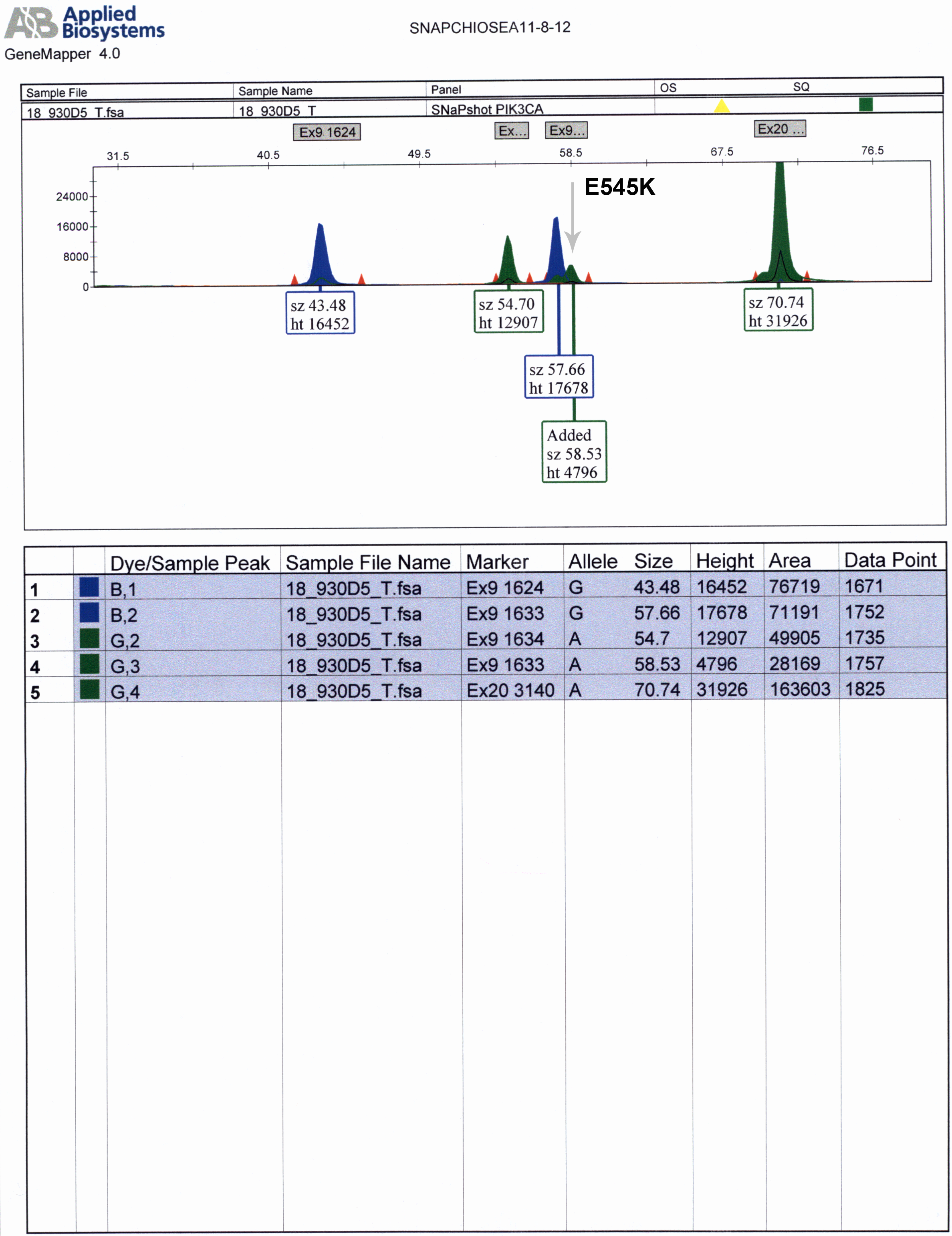

Supplement: Additional file 3: Figure S3 — SNaPshot detection of common hotspot mutations in the exon 9 of the PIK3CA gene. SNaPshot result illustrating c. 1633G>A, p.E545K electropherogram pattern (mutant peak is indicated by a grey arrow). The bases are color-coded: “A” – green, while “G” – blue. The red trigon-shaped peaks represent internal size standards. [file 1471-2407-13-602-S3.tiff]

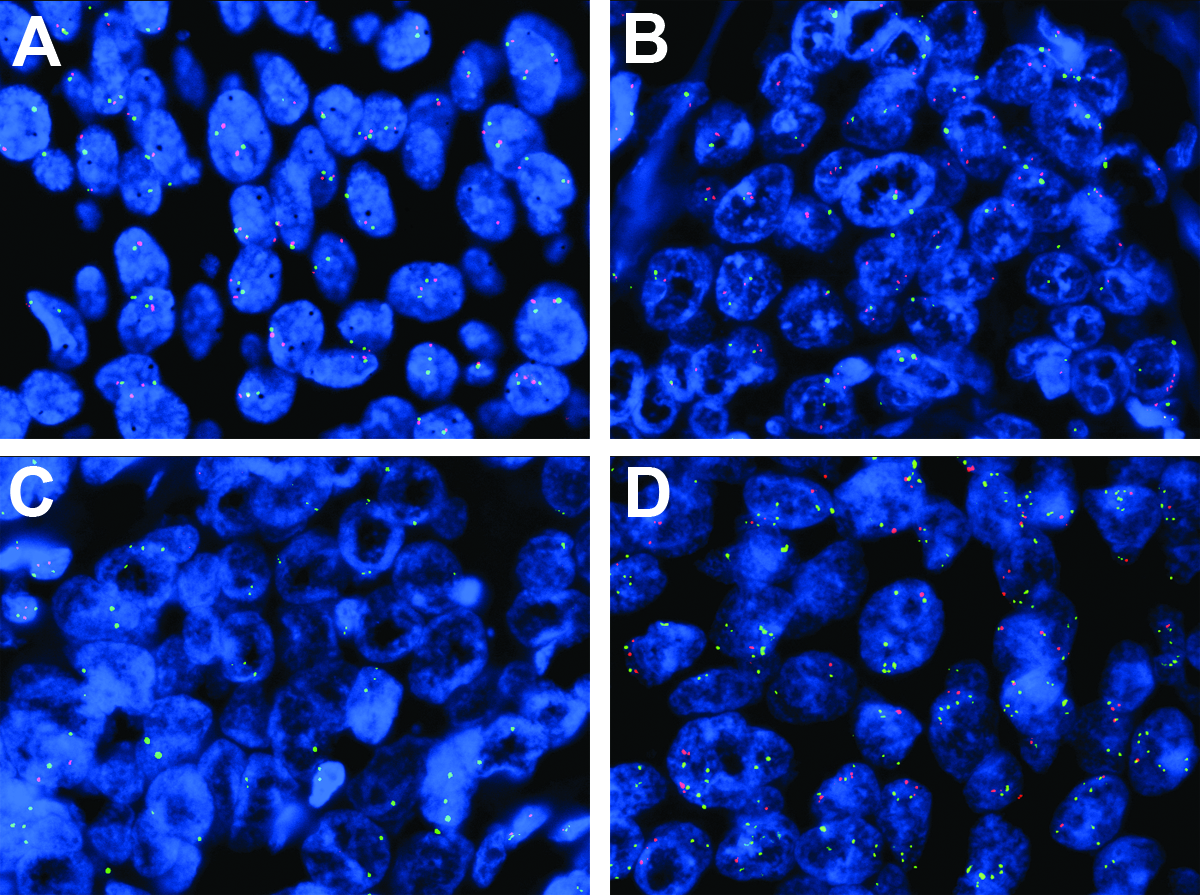

Supplement: Additional file 4: Figure S4 — Representative images of PTEN and PIK3CA fluorescence in situ hybridization, FISH, original magnification 1000x. All cases included in this figure are oropharyngeal squamous cell carcinoma that are human papillomavirus-positive, HRAS wild-type, PIK3CA wild-type, without PIK3CA amplification. The nuclei were counterstained with DAPI/Antifade 1 (blue) (Vysis, Inc., Downers Grove, IL). A. PTEN FISH, representative field: most nuclei are characterized by two PTEN signals (orange) and two chromosomal enumeration probe 10 (CEP10) signals (green), consistent with normal PTEN copy number. B. PTEN FISH, representative field: 57.1% of cells (36/63) showed only one pair of PTEN and CEP10 signals, consistent with chromosome 10 monosomy. C. PTEN FISH, representative field: 90.2% (55/61) of analyzed cells showed no PTEN signal, consistent with homozygous PTEN loss. Also, 55.7% (34/61) of analyzed cells showed only one CEP 10 signal consistent with chromosome 10 monosomy. The PTEN/CEP10 ratio was 0.11. D. PIK3CA (green) and CEP3 (orange) FISH, representative field. The PIK3CA/CEP3 ratio is 2.5. [file 1471-2407-13-602-S4.tiff]
